# Pathology of Equine Influenza virus (H3N8) in Murine Model

**DOI:** 10.1371/journal.pone.0143094

**Published:** 2015-11-20

**Authors:** Selvaraj Pavulraj, Bidhan Chandra Bera, Alok Joshi, Taruna Anand, Meenakshi Virmani, Rajesh Kumar Vaid, Karuppusamy Shanmugasundaram, Baldev Raj Gulati, K. Rajukumar, Rajendra Singh, Jyoti Misri, Raj Kumar Singh, Bhupendra Nath Tripathi, Nitin Virmani

**Affiliations:** 1 National Research Centre on Equines, Hisar, Haryana, India; 2 Veterinary Hospital—Naini, Barakot, Almora, Uttarakhand, India; 3 Department of Veterinary Physiology and Biochemistry, Lala Lajpat Rai University of Veterinary & Animal Sciences, Hisar, Haryana, India; 4 National Institute of High Security Animal Diseases, Bhopal, MP, India; 5 Division of Pathology, Indian Veterinary Research Institute, Bareilly, UP, India; 6 Division of Animal Science, Krishi Bhavan, New Delhi, India; 7 Indian Veterinary Research Institute, Bareilly, UP, India; St. Jude Children's Research Hospital, UNITED STATES

## Abstract

Equine influenza viruses (EIV)—H3N8 continue to circulate in equine population throughout the world. They evolve by the process of antigenic drift that leads to substantial change in the antigenicity of the virus, thereby necessitating substitution of virus strain in the vaccines. This requires frequent testing of the new vaccines in the *in vivo* system; however, lack of an appropriate laboratory animal challenge model for testing protective efficacy of equine influenza vaccine candidates hinders the screening of new vaccines and other therapeutic approaches. In the present investigation, BALB/c mouse were explored for suitability for conducting pathogenecity studies for EIV. The BALB/c mice were inoculated intranasally @ 2×10^6.24^ EID_50_ with EIV (H3N8) belonging to Clade 2 of Florida sublineage and monitored for setting up of infection and associated parameters. All mice inoculated with EIV exhibited clinical signs *viz*. loss in body weights, lethargy, dyspnea, etc, between 3 and 5 days which commensurate with lesions observed in the respiratory tract including rhinitis, tracheitis, bronchitis, bronchiolitis, alveolitis and diffuse interstitial pneumonia. Transmission electron microscopy, immunohistochemistry, virus quantification through titration and qRT-PCR demonstrated active viral infection in the upper and lower respiratory tract. Serology revealed rise in serum lactate dehydrogenase levels along with sero-conversion. The pattern of disease progression, pathological lesions and virus recovery from nasal washings and lungs in the present investigations in mice were comparable to natural and experimental EIV infection in equines. The findings establish BALB/c mice as small animal model for studying EIV (H3N8) infection and will have immense potential for dissecting viral pathogenesis, vaccine efficacy studies, preliminary screening of vaccine candidates and antiviral therapeutics against EIV.

## Introduction

Equine influenza (EI) is an OIE listed highly contagious acute respiratory disease of equines *viz*. horses, mules, donkeys and zebras caused by Influenza A virus. Equine influenza viruses (EIVs) are responsible for two third of viral respiratory infections in horses [1] and the disease is characterized by pyrexia, anorexia, depression, dyspnea, dry hacking cough, serous nasal discharge and secondary bacterial pneumonia [2]. In equines, two subtypes of virus *viz*. H7N7 and H3N8 have been reported to be responsible for disease, however, H7N7 subtype has not been isolated since 1980 [3,4]. H3N8 viruses continue to circulate in the equine population throughout the world and are essentially enzootic in USA and Western Europe. EIVs like any other influenza viruses constantly evolve by the process of antigenic drift as a result of point mutations in haemagglutinin (HA) and neuraminidase (NA) genes leading to substantial change in the antigenicity of the virus [5]. This causes failure of the vaccines as antibodies elicited against earlier strains do not protect against newly evolved variants. The frequent antigenic drift in influenza viruses leads to changes in the antigenic structure, which warrants regular harmonization of vaccines either by new strain substitution or by developing a new vaccine altogether. The studies in homologous host (equines) for immunopathogenecity and potency testing are difficult, expensive and involve ethical issues. Although ferrets have been widely used as a model, lack of their availability worldwide with regard to inbred lines, specific pathogen free status, size, cost, requirement of specific housing, labour, intensive handling, natural ability to acquire other influenza virus infection [6] and lack of ferret specific biologicals make them difficult model for vaccine efficacy testing and routine EIV research. Ideally small animal model should mimic disease progression and pattern observed in the natural host. Mice have been used widely as small animal model for influenza virus research including those with H1N1, H3N2, H5N1, H7N3, H7N7, H7N2, H2N9 and H7N9 [7–13]. The susceptibility of mice to influenza viruses depends on both the strain of mice and strain of influenza viruses. Common strains of mice used for influenza research are either BALB/C or C57BL/6 [14]. Lack of functional Mx1 protein, a critical antiviral factor in inbred laboratory mice make them susceptible to influenza virus infection [15]. Certain influenza viruses cause disease in mice without prior adaptation like 1918 H1N1 pandemic strain [16], highly pathogenic avian influenza (HPAI) viruses of H5N1 subtypes [17], H7N7 viruses isolated from humans [18] and certain low pathogenic avian influenza (LPAI) virus subsets [12]. Further, an inbred strain- DBA/2 has been explored for influenza virus studies (H5N1, H3N2, H1N1, mice adopted PR/8, etc.) and found to be more susceptible without adaptation of virus as indicated by weight loss, severe form of disease and mortality [19, 20]. However, avian viruses that are low pathogenic in natural host are highly pathogenic in DBA/2 mice [21]. Increased pathology in DBA/2 mice has been correlated with enhanced inflammatory response [22] which warrants further studies to reveal the reason for differential host response in DBA/2 mice at genetic and molecular level to understand host-pathogen causal relationship.

It has been established that avian and equine influenza viruses attach preferentially to sialic acid moieties with an α-2, 3 linkages, which are predominant in mouse respiratory tract [23]. In past, increase in virulence of the influenza H7N7 viruses isolated from equine has been observed after serial passage in mice [24]. In a previous study, H3N8 virus isolated from dogs (A/canine/Florida/04) (canine/FL/04) and an old EI virus (A/equine/Kentucky/91) (eq/KY/91) were compared for the development of lesions in respiratory tract of BALB/c mice, however, the intensity of lesions with equine virus was of very less in severity [25]. Experiment carried out in BALB/c mice with predivergent (A/equine/Romania/1/80 (eq/ROM/80) and A/equine/Georgia/1/81 (eq/GA/81)) and Clade 2 (A/equine/Newmarket/5/03 (eq/NM/03)) H3N8 EIVs revealed that all 3 strains replicated to high titre in mice upper and lower respiratory tract between 2–4 days post infection (dpi), and elicited neutralizing antibodies against homologous virus and cross neutralizing antibody response to heterologous EIVs in the study (except eq/NM/03), however none of mice developed significant weight loss or lethality [26]. Recently, live cold adapted (ca) reverse genetically engineered H3N8 virus vaccine bearing HA and NA genes from predivergent EIV (eq/GA/81) conferred complete protection against homologous and heterologous H3N8 wild type virus challenge in mice as indicated by no virus detection in vaccinated mice [27]. There are no reports on detailed pathological investigations of EIV (H3N8) infection in mice. The present studies were thus undertaken to develop a mouse model for equine influenza (H3N8) infection which shall have potential for studying molecular viral pathogenesis, virus-host interactions, preliminary screening of vaccines, antiviral drugs and therapeutics prior to final testing in the natural host, reverse genetics genomic studies, etc.

## Materials and Methods

### Ethics and bio-safety statement

Approval for animal experimentations were obtained from Institute Animal Ethical Committee (vide approval no.NRCE/CPCSEA/193) and Institute Bio-safety Committee (4^th^ IBSC meeting held on 17.02.2014) of National Research Center on Equines (NRCE), Haryana, India. As the experiment was aimed to study the clinical response and pathology of EI in BALB/c mice, use of analgesics was expected to mask or alleviate the actual clinical signs and response of mice to infection and thus IAEC approval for non usage of specific measures to reduce pain or sufferings following EIV infection in BALB/c mice was taken. Nonetheless, mice were acclimatized, *ad libitum* feed and water was provided and housed in micro-ventilator cages in Bio-safety Level-3 (BSL) facility at NRCE, Hisar, Haryana, India as per the guidelines defined by the Committee for the Purpose of Control and Supervision of Experiments on animals (CPCSEA), Ministry of Environment and Forestry, Government of India. The permission for using embryonated chicken egg for virus isolation is not required in India, however necessary approval for animal experimentations have been taken as mentioned above. Infected tissues and other bio-waste materials were safely disposed via private partner (Synergy Waste Management [p] limited, Hisar, India).

### Equine influenza virus (H3N8) propagation

The EIV {A/equine/Jammu-Katra/08 (H3N8; eq/J-K/08)} isolated from 2008–09 outbreak in India belonging to Clade 2 of Florida sublineage [28, 29] and maintained at passage three in the allantoic cavity of 9–11 days old embryonated chicken eggs in Equine Pathology Laboratory at NRCE, Hisar was used in this study. The selected EIV is genetically very close (99%) to Florida Clade 2 virus- A/equine/Richmond/07- which is recommended for purpose of vaccination by OIE Expert Surveillance panel for equine influenza virus strain selection (http://www.oie.int/our-scientific-expertise/specific-information-and-recommendations/equine-influenza/). The virus was propagated in 9–11 days old embryonated chicken egg obtained from Government Hatchery, Hisar, Haryana, India, through allantoic cavity route and harvested allantoic fluid was stored at -70°C to produce stock of EIV to infect mice. Quantification of EIV in stock virus culture was performed by calculation of 50% egg infectious dose (EID_50_) using Reed and Muench method (1938) [30]. Briefly, a serial 10 fold dilution of stock was prepared in PBS up to 10^−10^ dilution. Three embryonated eggs were inoculated with 100μl per egg per dilution and incubated at 34°C for 72 hrs. Haemagglutination (HA) assay was performed on harvested allantoic fluid as described by Hirst (1941) [31]. Reed and Muench method (1938) [30] was used to calculate 50% end point result and expressed as EID_50_. Allantoic fluid harvested from healthy embryonated chicken eggs was utilized for mock inoculation of negative control mice.

### Intranasal inoculation in BALB/c mice

BALB/c mice (n = 72), 4–5 weeks of age of either sex, were procured from Institute of Microbial Technology, Chandigarh, India and housed in micro-ventilator cages under BSL-3 facility before experimental inoculation. Mice were weighed and routine health care management was practiced daily. Mice were divided into two groups *viz*. Group I—EIV inoculated (n = 48) and Group II—mock inoculated (n = 24). Prior to experimental EIV infection, mice were anesthetized with Xylazine and Ketamine mixture (10mg of xylazine + 50mg of ketamine) @ 100mg/kg body weight intra-peritoneally. Group I mice were inoculated with 2×10^6.24^ EID_50_ EIV (H3N8) in 20μl volume through intranasal route, while group II mice were mock inoculated with 20μl of allantoic fluid harvested from healthy embryonated chicken egg.

### Clinical signs and post mortem examination

Mice were monitored closely for the presence of clinical signs and bodyweight loss. EIV inoculated mice (n = 6) and mock inoculated mice (n = 3) were euthanized at intervals—12 hpi, 1, 2, 3, 5 7, 10 and 14 dpi by cervical dislocation. Internal organs were examined for presence of gross lesions. Blood, serum and tissue samples were collected for further analysis.

### Serum biochemistry

Biochemical analysis of pooled mice serum samples (n = 6) at each interval from respective groups was performed using ERBA-EM-200® biochemical analyzer. Serum level of lactate dehydrogenase (LDH), SGPT, SGOT, cholesterol, alkaline phosphatase, Gamma GT, creatine kinase, phosphorus, total bilirubin, urea, creatinine, glucose, triglyceride, high density cholesterol, low density cholesterol, uric acid, calcium, albumin and total proteins were estimated.

### Serology

Haemagglutination inhibition (HAI) assay was performed in microtitre plates on serum samples collected from mice at various intervals as per OIE protocol (2012) [4]. Briefly, the serum samples were pretreated with 0.016 M potassium periodate in PBS and excess periodate solution was neutralized by 3% glycerol in PBS, further heat inactivated at 56°C for 30 min. Ether treated EIV antigen (eq/J-K/08)[4 HA units] was used for assay.

### Histopathology

Representative tissue samples *viz*. nasal turbinate (half), trachea, lung (left lobe), heart, liver, kidneys, spleen, brain, stomach, intestine, pancreas, lymph nodes, etc were collected from each mouse and immersion-fixed in 10% neutral buffered formalin for at least 96 hrs. Further, specimens were processed by conventional methods, embedded in paraffin, sectioned at 3–4 μm thickness and stained with hematoxylin and eosin (H&E) as described by Luna (1968) [32]. Stained slides were examined under the microscope (Nikon model-81i).

### Immunohistochemistry

To confirm active viral infection in mice and for localization of viral antigens in tissues, immunohistochemical staining of tissue sections using hyper immune serum directed against whole EIV in rabbit (at NRCE, Hisar) was performed. Briefly, re-hydrated tissue sections were placed in 3% H_2_O_2_ solution in methanol for antigen retrieval followed by treatment with trypsin (1mg/ml) and calcium chloride solution (1mg/ml) for 30 min at 37°C. Further, the sections were blocked with 4% skimmed milk in TBS for 1 hr at 37°C. The sections were exposed to primary rabbit hyper immune serum raised against EIV (HAI titre 1:128) at the dilution of 1:60 for 1 hr at 37°C followed by treating the sections with horse radish peroxidase—labeled anti-rabbit IgG raised in goat (Sigma® A-9169). Immuno-reactivity was detected using a 3, 3’-diaminobenzidine tetrahydrochloride (Sigma® D5905) substrate and H_2_O_2_. Sections were counterstained with 10% Harris Haematoxylin stain and examined under microscope.

### Transmission electron microscopy

Electron microscopic studies on mice tissues were performed at National Institute of High Security Animal Diseases (NIHSAD), Bhopal. Trachea and lung tissues (1 mm × 1 mm size) collected from mice were immersion-fixed in 2.5% glutaraldehyde solution in phosphate buffer at 4°C for 12 hrs. Following dehydration and clearing, fixed tissues were embedded in Araldite 502®. Semi-thin sections of 400 nm thickness were cut using an ultra microtome (Ultracut, Leica®, USA) and stained with multiple staining solution (Polysciences Inc., USA). The stained sections were examined under light microscope to identify the area of interest for ultrathin sectioning for TEM. Ultra thin sections of 60 to 90 nm were cut using ultra microtome. Sections were stained with saturated uranium acetate and lead citrate solution. Grid examination for ultrastructural changes was carried out on a transmission electron microscope (JEM-1400®, Jeol, Japan).

### Virus isolation

Replication of EIV in mice respiratory tract was examined by determining the virus titre in nasal wash solution and lung tissue collected at different intervals. Nasal washings were collected from mice after sacrifice by tying trachea towards trachea-bronchial junction and injecting 300μl of HBSS containing antibiotic and antifungal solution. The nasal wash was collected from external nares and stored at -70°C. For virus isolation and titration, lung tissue (25mg) was homogenized in cold PBS followed by serial 10-fold dilutions and inoculation in embryonated chicken egg through intra-allantoic route. EIV titre in harvested allantoic fluid was estimated by Reed and Muench (1938) [30] method and expressed as EID_50_ per 25mg of lung tissue or 140μl nasal wash solution.

### Quantification by Real-time RT-PCR

RNA from lung homogenate (25 mg), nasal washings (140 μl) and stock virus was extracted using QIAamp® Viral RNA Mini kit (Qiagen, Valencia, CA) as per the manufacturer’s instructions. The quality and quantity of the isolated RNA was checked in BioPhotometer Plus® (Eppendorf) and stored at -80°C for further analysis by qRT-PCR.

TaqMan probe based quantitative RT-PCR was performed for estimation of viral loads in terms of copy numbers in lung homogenate and pooled nasal washing samples collected at various time intervals *viz*.,12 hrs, 1, 3, 5, 7, 10 and 14 dpi. qRT-PCR was performed using primers [Forward primer (EqFlu NP F): GAAGGGCGGCTGATTCAGA; Reverse primer (EqFlu NP R): TTCGTCGAATGCCG AAAGTAC] and probe (EqFlu NP Prb: FAM-CAGCATAACAATAGAAAGGA- BHQ1) which were specific to conserved region of nucleoprotein (NP) gene of influenza A virus as described by Lu *et al*. (2009) [33]. The assay was carried out employing TaqMan® Fast Virus 1-Step Master Mix (Applied Biosystems) in Step-One Real-Time PCR Machine (Applied Biosystems). The reporter dye-FAM was incorporated in the synthesized probe for compatibility of the emission spectra detected in Real-Time PCR machine. Four standards of known quantity (10^5^ to 10^2^ copies) of *in vitro* transcribed (IVT) RNA of cloned NP gene were included in every reaction set up for quantification of EIVs. The 10 μl reaction mixture contained 2 μl of extracted RNA, 2.5 μl of 4xTaqMan® Fast Virus 1- Step Master Mix, 5 μM of EqFlu NP probe (0.5 μl), 10 μM of forward and reverse primers (0.9 μl each) and 3.2 μl nuclease free water for each sample analyzed. The Fast mode cycling condition was run in Real-Time PCR machine in following thermal profile: hold for 5 min at 50°C and 20 sec at 95°C followed by 40 cycles of amplification (95°C for 3 sec and at 60°C for 30 sec). Automatic threshold for the Ct was selected and the results were analysed following standard curve with the positive standards of efficiency >95% and R^2^ value >0.980.

### Statistical analysis

The data were expressed as means ± SEM and subjected to statistical analyses using GraphPad PRISM® software version 5.04. The two-way ANOVA was followed by Bonferroni post-hoc tests to compare replicate means by row. A value of p<0.05 was considered statistically significant.

## Results

### Clinical signs and body weight

**Fig 1:**
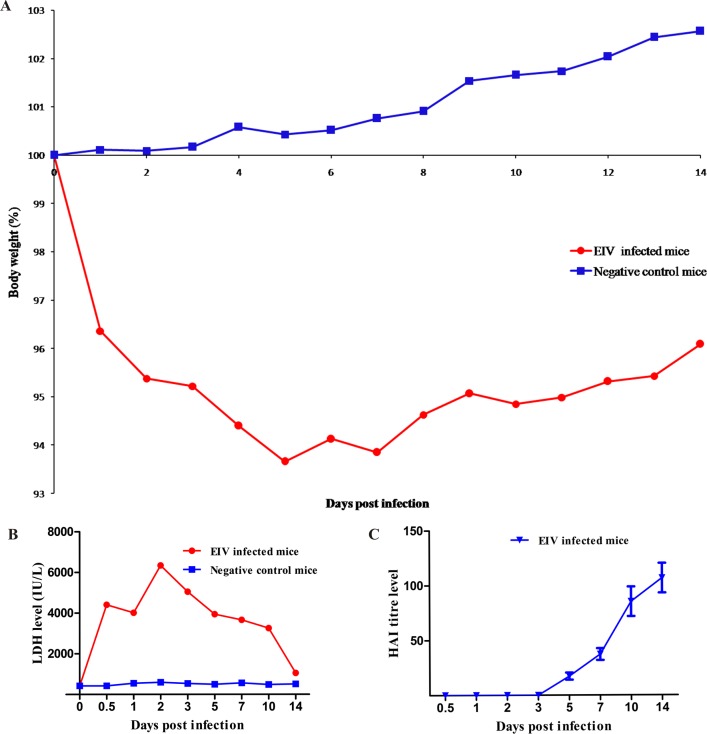
Pathogenicity of eq/J-K/08 H3N8 EIV in mice: **A:**Percentage changes in body weight of EIV infected mice and negative control mice during the period of experiment (0–14 dpi). **B:**LDH level in pooled serum samples from EIV infected mice during the period of experiment. **C:**Humaral immune response following experimental EIV infection in BALB/c mice.

BALB/c mice inoculated with EIV (group I) began to show respiratory distress and crouching at corners from 2 dpi (days post infection) onwards. The clinical signs were characterized by lethargy, forced expiration, ruffled coat, decreased activity and crouching at the corners with reduced feed and water intake between 3 and 5 dpi. Severity of the clinical signs increased up to 7 dpi followed by a progressive decline and no clinical signs were observed at 14 dpi. Appearance of clinical signs in mice was in concurrence with reduction in body weights. Maximum weight reduction was observed on 5 dpi (6.34±0.21%) (Fig 1A). Mice started regaining body weight 8 dpi onwards but failed to reach to original body weight till the end of the experiment i.e. 14 dpi (S1 Table). No mortality was observed in the infected mice. None of the mice from mock inoculated group (group II) showed any clinical signs or significant changes in the body weight.

### Serum biochemistry

Except for the level of lactate dehydrogenase (LDH), all serum biochemical parameters were within the normal range. Elevated serum LDH enzyme was observed as early as 12 hours post infection (hpi) with mean level of 4413 IU/L and peaked at 2 dpi (6344 IU/L) (Fig 1B). LDH levels decreased to basal level by the end of experiment (14 dpi) (S3 Table). Serum biochemical profiles of all mice in the control group were in normal range during the entire period of study.

### Humoral immune response

None of the mock infected mice showed any haemagglutination inhibition (HAI) antibody titre. The EIV inoculated mice had no detectable levels of HAI antibody titre till 3 dpi, but the titre rose steadily from 5 dpi (17.33±3.21) to 14 dpi (106.67±13.49) dpi (Fig 1C). All mice were sero-negative to EI by HAI before experimental inoculation (S2 Table).

### Gross findings

**Fig 2:**
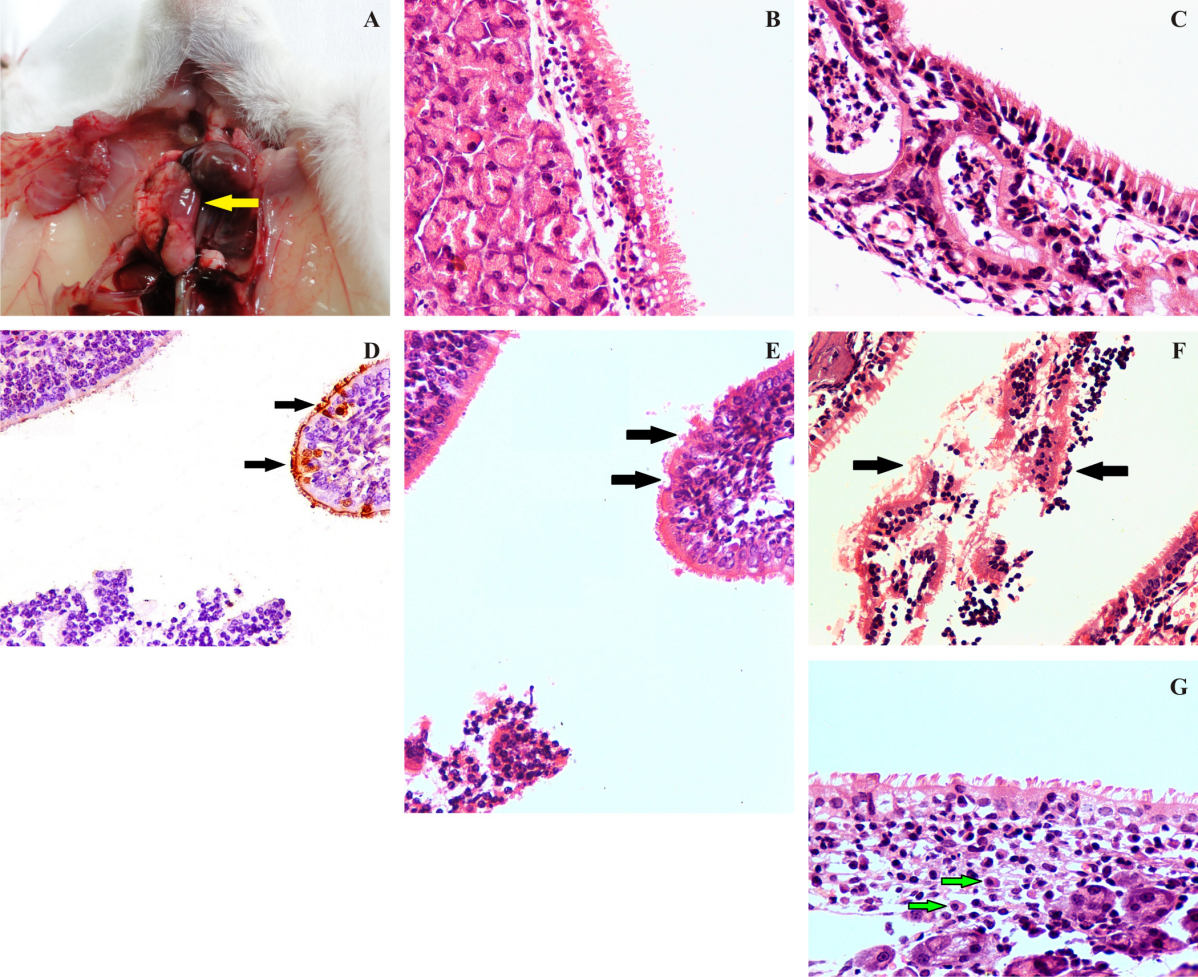
Pathogenicity of eq/J-K/08 H3N8 EIV in mice: **A:**Focal area of consolidation (red hepatization—arrow) of lung with moderate congestion at 5 dpi (n = 6). **B:** Degeneration of lining epithelial cells with diffuse goblet cell hyperplasia at 2 dpi X400. **C:** Degeneration of lining epithelial cells, severe degeneration of submucosal glandular epithelium and impacted lumen with neutrophils and necrotic tissue debris 3 dpi X400. **D:** Loss of cilia, with degeneration and necrosis of lining epithelium (arrow) of nasal mucosa at 2 dpi. **E:** Immunohistochemical staining (Fig 2C section) for EIV antigens in cytoplasm and nucleus of degenerated and necrotic lining epithelial cells of nasal turbinate (arrow) at 2 dpi (IIPT) X400. **F:** Severe degeneration of lining epithelial cells with goblet cell hyperplasia and presence of denuded ciliated epithelium along with lymphocytes in lumen (arrow) at 5 dpi X400. **G:** Goblet cell hyperplasia of lining epithelial cells with infiltrations of macrophages (arrow) and lymphocytes in lamina propria at 5 dpi X600 (n = 6).

Gross lesions in the EI infected mice were confined mainly to the lower respiratory tract. Lung lesions characterized by red hepatization around hilus could be appreciated from 1 dpi onwards. At 2 and 3 dpi, lungs showed larger area of red hepatization, consolidation (3–4 mm × 2–3 mm) and mild gray discoloration of the lobes. Areas of consolidation were focal and well demarcated from the surrounding parenchyma. Mucosa of the nasal turbinate revealed moderate congestion, and trachea contained grayish mucinous exudate in the lumen. By 5 dpi, all lung lobes were grayish with areas of severe consolidation (>4 mm × 3 mm) of parenchyma and transition of red hepatization to gray hepatization (Fig 2A). Lesions were moderately severe at 7 dpi and comprised of gray hepatization of consolidated lung parenchyma and grayish discoloration of rest of the parenchyma. Lesions became less severe by 10 dpi and no distinct gross lesions could be observed at 14 dpi. Mock inoculated mice (Group II) did not show any lung lesion throughout the period of the experiment.

### Histopathological findings

Histopathology and immunohistochemistry revealed localization of lesions and distribution of EIV antigen to nasal turbinates, trachea and lungs in the group I mice. The mock inoculated animals (group II mice) did not show any histological changes of pathological significance at various intervals. EIV antigen was detected in the nasal turbinate, tracheal, bronchial and bronchiolar epithelial cells and interstitial macrophages between 12 hpi to 5 dpi. The detail organ-specific lesions are depicted as follows:

#### Nasal turbinate

Lesions *viz*. degeneration and denudation of the lining epithelium, congestion of submucosal blood vessels, impacted nasal turbinate lumen with inflammatory exudates comprising of neutrophils and lymphocytes, denuded ciliated epithelial cells and necrotic tissue debris mixed with mucus could be appreciated on 2 and 3 dpi in the nasal turbinates (Fig 2B, 2C and 2D). EIV antigen could be demonstrated in the sections showing necrosis of epithelial lining cells (Fig 2D and 2E). At 5 dpi, moderate degeneration and sloughing of lining epithelial cells and infiltration of lymphocytes in lamina propria with severe congestion of the blood vessels was observed (Fig 2F and 2G). No appreciable lesions could be observed in the nasal turbinates after 7 dpi.

#### Trachea

**Fig 3:**
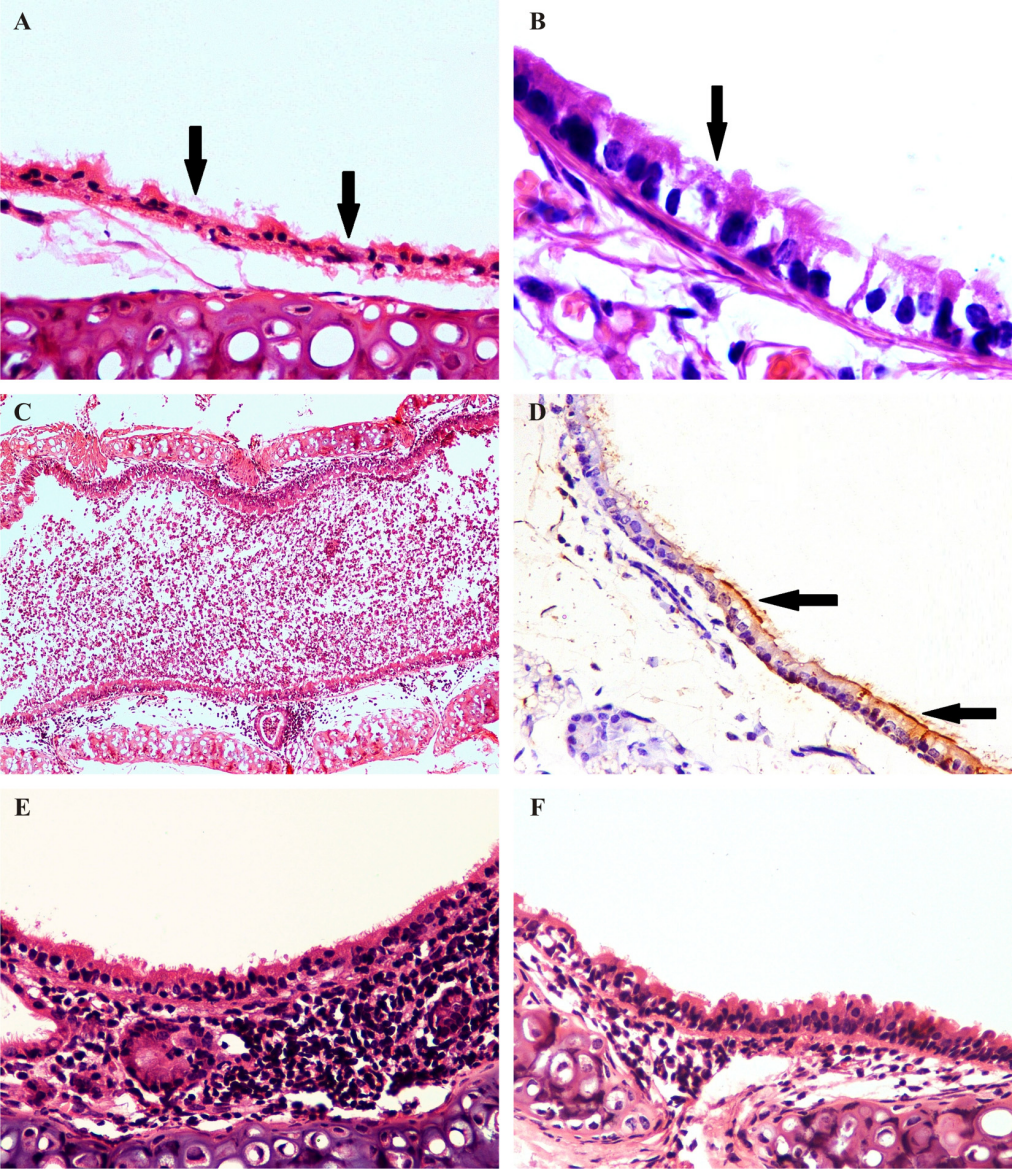
Pathogenicity of eq/J-K/08 H3N8 EIV in mice:Trachea (H&E): **A:** Severe degeneration and necrosis of lining epithelial cells (arrow) at 2 dpi X400. **B:** Loss of cilia with necrosis of mucosal lining epithelium (arrow) at 2 dpi X1000. **C:** Tracheal lumen completely impacted with denuded epithelial cells with inflammatory exudates mixed with mucus at 2 dpi X100. **D:** Immunohistochemical staining for EIV positive antigens in cytoplasm of lining epithelial cells (arrow) at 3 dpi (IIPT) X600.E: Moderate degeneration of lining epithelial cells with severe infiltration of lymphocytes, macrophages and neutrophils in lamina propria at 5 dpi X400. **F:** Regeneration of lining epithelial cells with mild infiltrations of macrophages in lamina propria X400 at 7 dpi (n = 6).

Severe tracheal lesions were observed in the EIV infected mice. The lesions were characterized by degeneration of lining epithelium with mild infiltration of granulocytes in lamina propria as early as 12 hpi. At 2 and 3 dpi, tracheal lumen was filled with necrotic tissue debris mixed with macrophages, neutrophils and denuded epithelial cells along with infiltration of macrophages in the lamina propria (Fig 3A, 3B and 3C). Distributions of EIV antigens could be observed in lining epithelial cells and macrophages in lamina propria (Fig 3D). Flattening of the lining epithelial cells with moderate infiltration of lymphocytes, macrophages, plasma cells and neutrophils in the lamina propria were observed at 5 dpi (Fig 3E). Squamous metaplasia of the lining epithelium and lamina propria showing mild infiltration with lymphocytes and macrophages was observed between 7 and 10 dpi (Fig 3F).

#### Lungs

**Fig 4:**
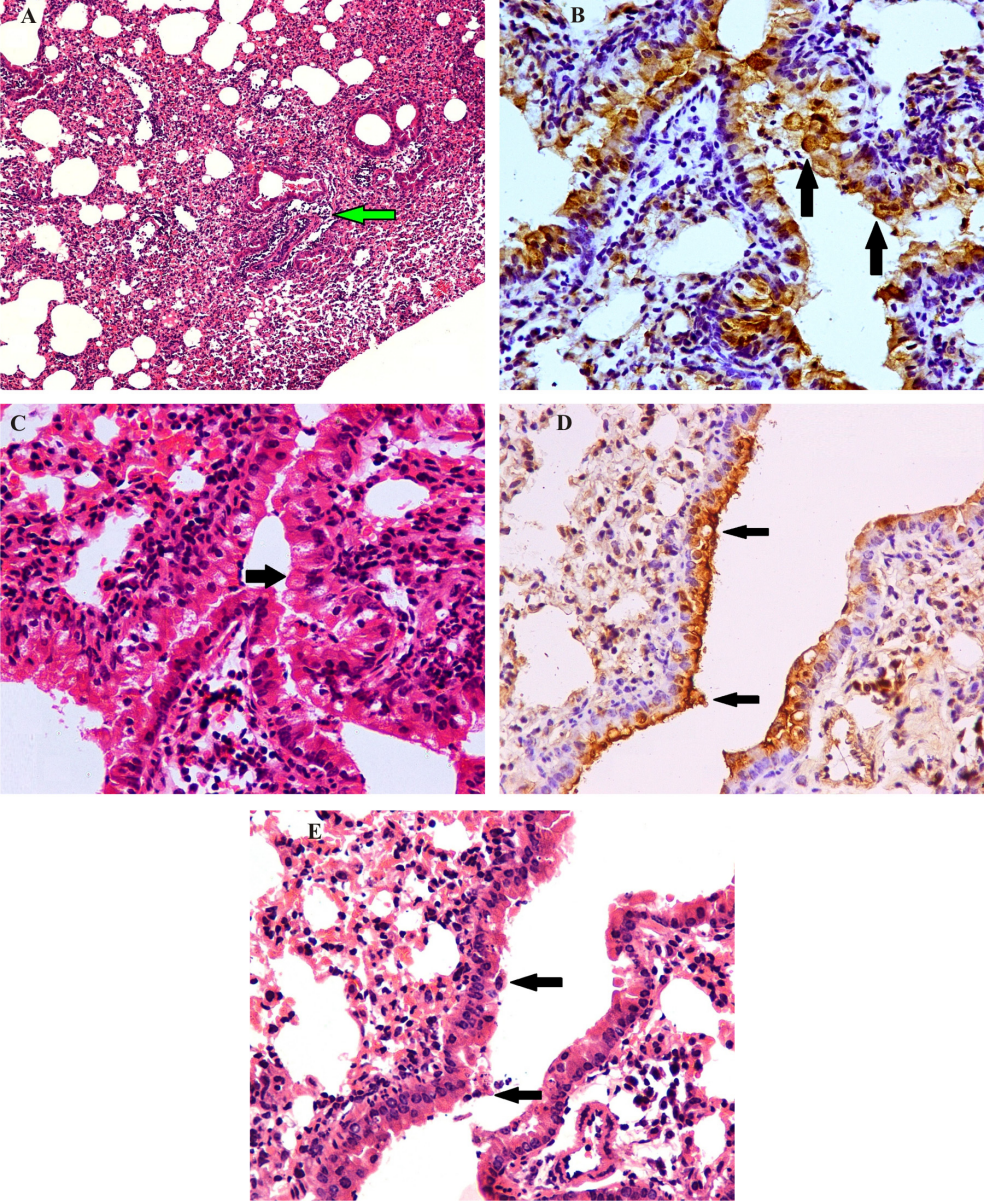
Pathogenicity of eq/J-K/08 H3N8 EIV in mice:Lung (H&E): **A:** Early interstitial thickening with mild perivascular cuffing by inflammatory cells (arrow) at 1 dpi X100. **B:** Majority of bronchial epithelial cells shows hydropic degeneration and necrosis of lining epithelial cells (arrow) at 12hpi. **C:** Degenerated and necrosed bronchial epithelial cells (Fig 4C section) reveals EIV positive antigens in cytoplasm (arrow) at 12 hr post infection (IIPT) X400 (n = 6). **D:** Moderate degeneration and denudation of bronchial lining epithelial cells (arrow) with macrophage infiltration in interstitial spaces at 2 dpi X400. **E:** Immunohistochemical staining for EIV positive antigens (Fig 4D section) in degenerated and denuded bronchiolar epithelial cells (arrow) and interstitial macrophages at 2 dpi (IIPT) X400 (n = 6).

Histopathological changes in lungs of the infected mice began to appear as soon as 12 hpi. Major changes were impacted bronchial and bronchiolar lumina with granulocytes, sloughed epithelial cells, eosinophilic necrotic tissue debris and mucus. Bronchiolar epithelial lesions were severe at 1 dpi and characterized by marked degeneration, blebing of cytoplasm, necrosis of bronchial and bronchiolar epithelium (Fig 4A). Moderate to severe vacuolar degeneration and mild necrosis of bronchial and bronchiolar epithelial cells at hilus and almost healthy alveoli were the prominent findings (Fig 4B). About 80% of lining epithelial cells were positive for EIV antigen (Fig 4C). Early consolidation was observed near the hilus. Margination and transmigration of neutrophils, lymphocytes and monocytes was observed in majority of blood vessels. The interstitial space was thickened due to diffuse infiltration of neutrophils, macrophages and necrotic tissue debris near larger bronchioles with mild BALT hyperplasia. Bronchial and bronchiolar epithelial cells and interstitial macrophages in the area of consolidations were markedly EIV positive.

**Fig 5:**
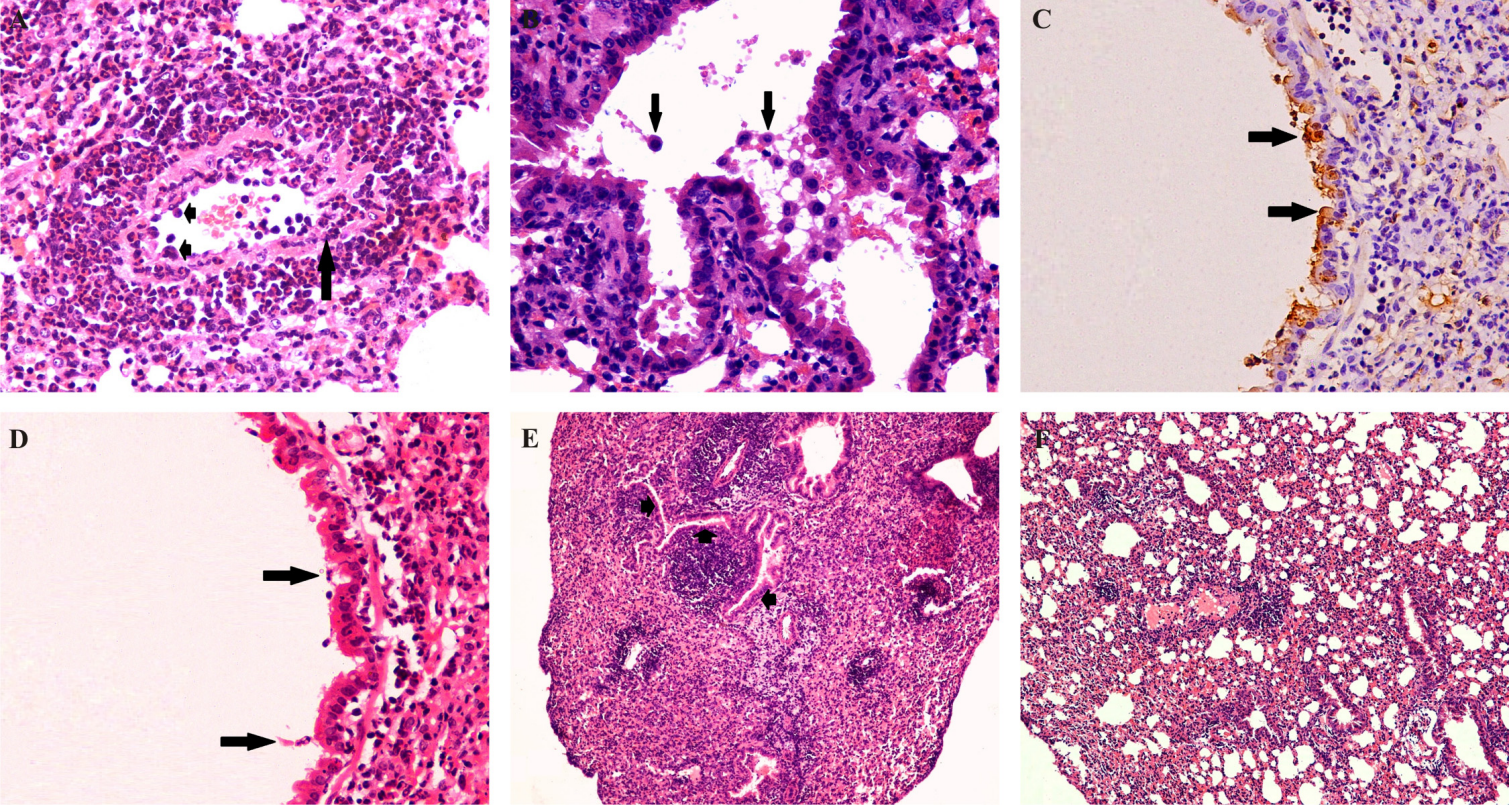
Pathogenicity of eq/J—K/08 H3N8 EIV in mice: Lung (H&E): **A:** Margination (arrow head), transmigration (arrow) and perivascular cuffing by neutrophils at 2 dpi X400. **B:** Necrotic bronchiolar epithelium with macrophages in bronchial lumen (arrow) at 5 dpi X400. **C:** Degeneration of lining epithelial cells (arrow) with mild peribronchiolar infiltration of lymphocytes at 5 dpi X400. **Immunohistochemical localization of EIV antigens in mice lung.** **D:** Distributions of EIV positive antigens in bronchiolar epithelial cells (arrow) at 5 dpi (Fig 5C section) (IIPT) X400 (n = 6). **E:** Severe consolidation of parenchyma with narrowing of bronchiolar lumen (arrow head) at 5 dpi X100 (n = 6). **F:** Resolution of lung lesion with mild thickening of alveolar septa and mild perivascular infiltrations at 14 dpi X100 (n = 6).

**Fig 6:**
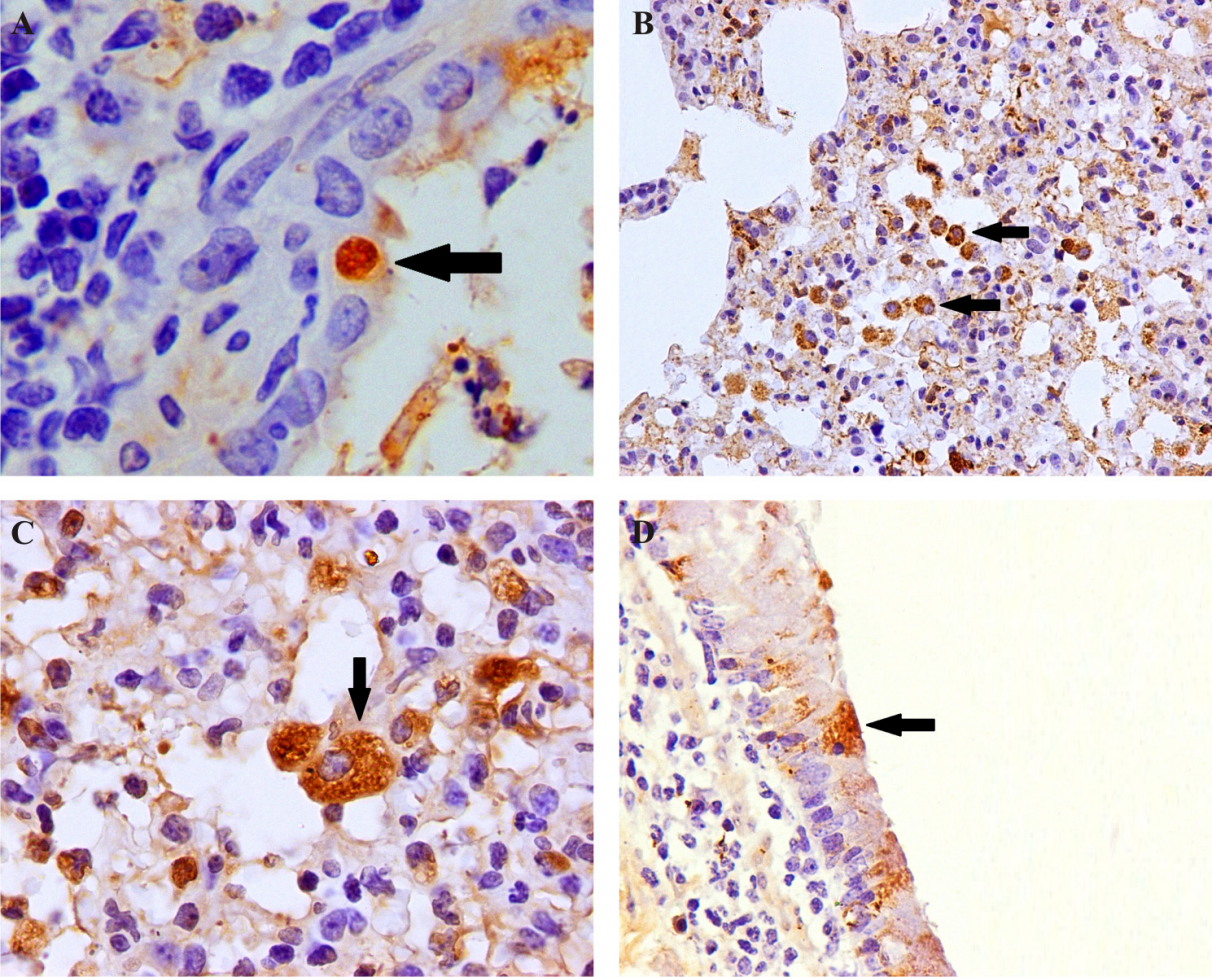
Pathogenicity of eq/J-K/08 H3N8 EIV in mice: **Immunohistochemical localization of EIV antigens in mice lung. A:** Intra-cytoplasmic EIV positive antigens in bronchiolar epithelial cells (arrow) at 3 dpi X1000. **B:** EIV positive interstitial macrophages (arrow) at 3 dpi X400. **C:** EIV positive antigens in cytoplasm of interstitial macrophages at 3 dpi (arrow) X1000 (n = 6). **D:** EIV positivity in bronchial lining epithelial cells (arrow) at 5 dpi (IIPT) X600 (n = 6).

Vascular and alveolar parenchymal changes were severe at 2 dpi and characterized by severe degeneration and necrosis of bronchial epithelium, margination, perivascular cuffing of neutrophils and vascular congestion along with EIV antigens distributions in bronchial epithelium (Fig 4D and 4E and Fig 5A). Most of alveolar and interstitial spaces were impacted with neutrophils, lymphocytes, macrophages and necrotic tissue debris with interstitial edema. Majority of alveolar and interstitial macrophages were EIV positive at 3 dpi (Fig 2F and Fig 6A, 6B and 6C). Switching of perivascular cellular infiltrations from neutrophils to lymphocytes and macrophages could be observed between 3 and 5 dpi. Area of pulmonary consolidation was increased with accumulation of inflammatory cells mainly neutrophils, macrophages and lymphocytes along with necrotic tissue debris and hemorrhage in the lung parenchyma. Severe pulmonary parenchyma consolidation (about 50–60%) was observed at 5 dpi along with areas of consolidation showing type II pneumocyte proliferation and interstitial edema (Fig 5E). Perivascular spaces were uniformly infiltrated with lymphocytes. Degenerated and denuded bronchial epithelial cells and inflammatory exudates were seen plugging the lumen in the larger bronchi and bronchioles (Fig 5B and 5C).

Lesions at 7 dpi were similar to 5dpi with moderate squamous metaplasia of bronchial lining epithelium, severe diffused interstitial pneumonia and proliferation of type II pneumocytes leading to narrowing and obliteration of bronchioles and alveoli. At 10 dpi, severe proliferation of type II pneumocytes and focal area of accumulation of neutrophils, macrophages in the interstitial spaces with mild edema were observed, while bronchial and bronchiolar changes were minimal. An overall reduction in the lung lesions was observed at 14 dpi and included mild thickening of interstitial spaces, focal infiltration of macrophages and lymphocytes in parenchyma with mild perivascular lymphocytic infiltrations while bronchi and bronchioles were almost normal (Fig 5F).

### Immunohistochemistry

Distribution of EIV antigen was confined to respiratory tract. Abundant EIV antigen was detected in the nasal turbinate, tracheal, bronchial and bronchiolar epithelial cells as early as 12 hpi. About 80% of these lining epithelial cells were positive for EIV antigen (Fig 4B). Majority of alveolar and interstitial macrophages were EIV positive in the early stage of infection (1–3 dpi) (Fig 6A, 6B and 6C). Bronchial and bronchiolar epithelial cells and interstitial macrophages in the area of consolidations were markedly EIV positive at 1 dpi. At 2 dpi, lining epithelium, macrophages in lamina propria and necrotic tissue exudate admixed with macrophages in trachea were positive for EIV antigen, while nasal turbinate epithelial cells showed localization of EIV antigen in few focal areas (Fig 2D, Fig 3D and Fig 4E). Between 3 and 5 dpi, decreasing intensity of EIV positivity in the bronchial epithelium was observed (Fig 5D and Fig 6D). Thereafter, no EIV positive signals could be observed in the tissue sections. Mock inoculated mice did not show any positive signals for EIV at any of the intervals.

### Transmission electron microscopy (TEM)

**Fig 7:**
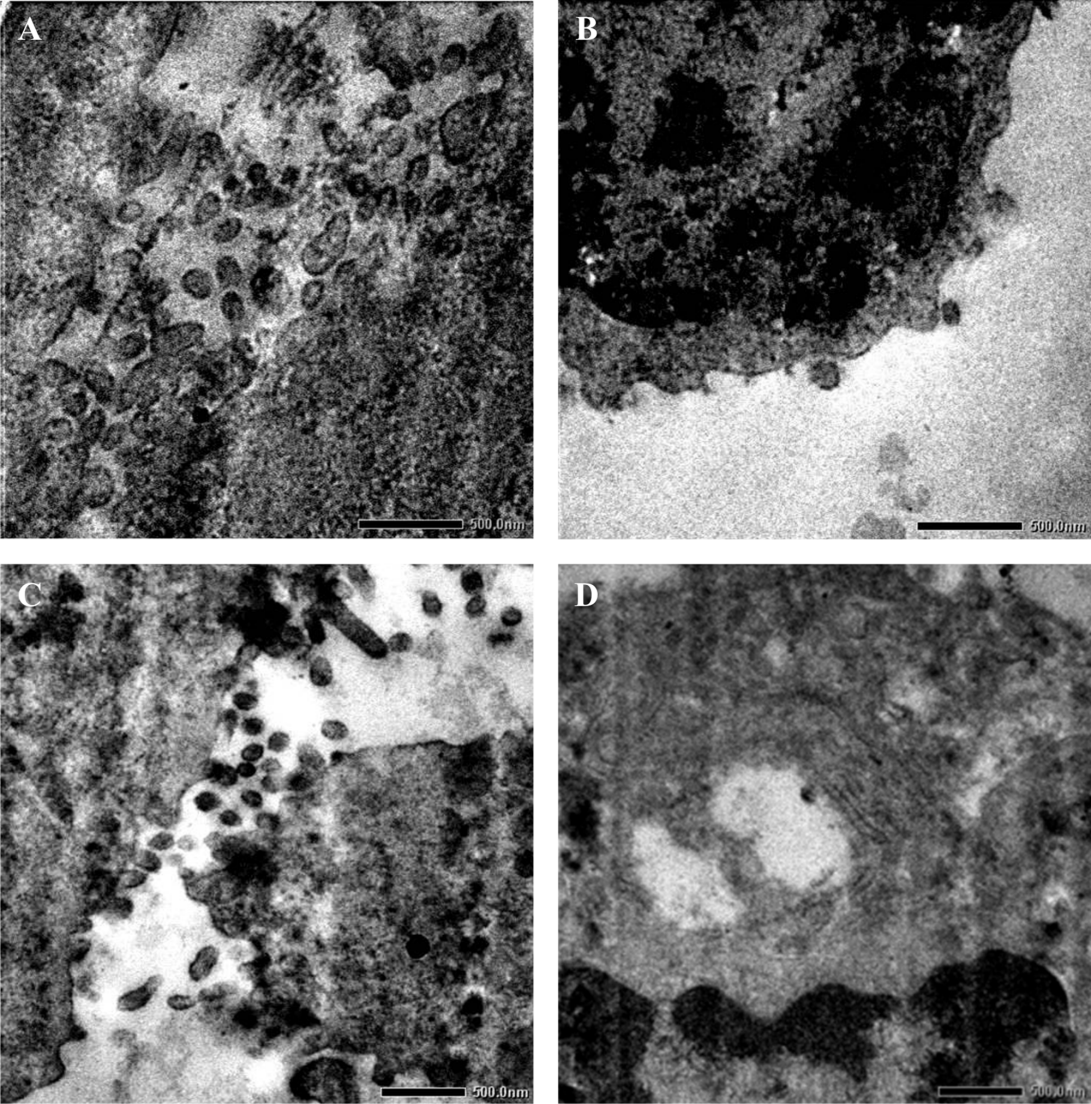
Pathogenicity of eq/J-K/08 H3N8 EIV in mice: **A:** Intercellular space with influenza virions, budding and released from surface of the degenerating cells from trachea at 3 dpi. Irregularly shaped microvilli can be seen amongst the virions X10000. **B:** Ultra thin section of lung at 3 dpi, showing budding of influenza virions from degenerating cell along with disintegration of nuclear envelope and loss of organelle architecture X 10000. **C:** Budding and release of influenza virions from surface of a degenerating cell in lung X8000. **D:** Fragmentation of nucleus with degeneration of endoplasmic reticulum of epithelial cells of lung at 3 dpi X8000 (n = 6).

TEM study of trachea revealed presence of influenza virions in the intercellular spaces characterized with budding and release of virions from the surface of degenerating cells at 3 dpi (Fig 7A). Presence of virions was observed among microvilli of epithelium. Degeneration of epithelial cells characterized by fusion of cilia, disintegration of mitochondria, endoplasmic reticulum and nucleus could be observed. Stained ultrathin sections of lungs showed presence of fully formed virion particles in the intercellular spaces along with budding from cell surface (Fig 7B). Spherical shaped influenza virions budding and released from degenerating cells were noticed along with presence of free influenza virions in cytoplasm of the degenerating cells (Fig 7C). Ultra structural lesions like disintegration of nuclear envelop, loss of organelle architecture (mitochondria and endoplasmic reticulum) with cytoplasm containing vesicles and autophagosomes could be observed (Fig 7D).

### Comparative pathology of EIV in BALB/c mice model and natural host

Referenced table on comparative pathology of EIV infection in BALB/c mice model from our experiment and natural or experimental EIV infection in equines are detailed in Table 1.

**Table 1:** Comparison of EIV infection in BALB/c mice and natural host.

S.No:	Disease pattern	Equines	BALB/c mice (present study)	References (for equines)
1.	Duration of virus shedding	5–6 days post infection(up to 7–10 days in some cases)	Up to 5 days post infection	[34–38]
2.	Mortality	Rare (common in young foals) associated with secondary bacterial complications	Not observed	[39, 40]
3.	Organs involved	Upper and lower respiratory tract	Similar to equines	[41]
4.	Serum biochemical changes	Not reported	Increases LDH level	
5.	Gross changes at necropsy	Hyperaemic nasal mucosa, petechial hemorrhages and mucopurulent exudates in trachea and bronchi, focal pulmonary hepatization in lungs	Congestion of nasal mucosa, mucinous exudates in trachea, multifocal parenchymal hepatization and consolidation in lungs	[34, 42]
6.	Histological changes	Rhinitis and tracheitis—diffuse degeneration, loss of cilia and necrosis of lining epithelium. Infiltrations of lymphocytes and macrophages in lamina propria.	Similar observations along with impactions of nasal and tracheal lumen with inflammatory exudates.	[34, 40, 42]
		Pulmonary hepatization—broncho-interstitial pneumonia with extensive neutrophilic infiltrations and proliferation of alveolar macrophages and type II pneumocytes.	Similar to broncho-interstitial pneumonic changes of equines at multi-focal areas along with BALT hyperplasia, perivascular cuffing by neutrophils, lymphocytes and monocytes.	
		Epithelial hyperplasia or squamous metaplasia of nasal, tracheal and bronchial epithelium.	Squamous metaplasia of tracheal and bronchial epithelium.	
7	EIV antigen distributions	Nasal mucosa, trachea, bronchial and bronchiolar epithelial cells and macrophages of lamina propria except alveolar macrophages	Lining epithelium of nasal turbinate, trachea, bronchi, bronchiole and macrophages in lamina propria and interstitial and alveolar spaces	[34]

### Virus isolation studies in embryonated chicken eggs

**Fig 8:**
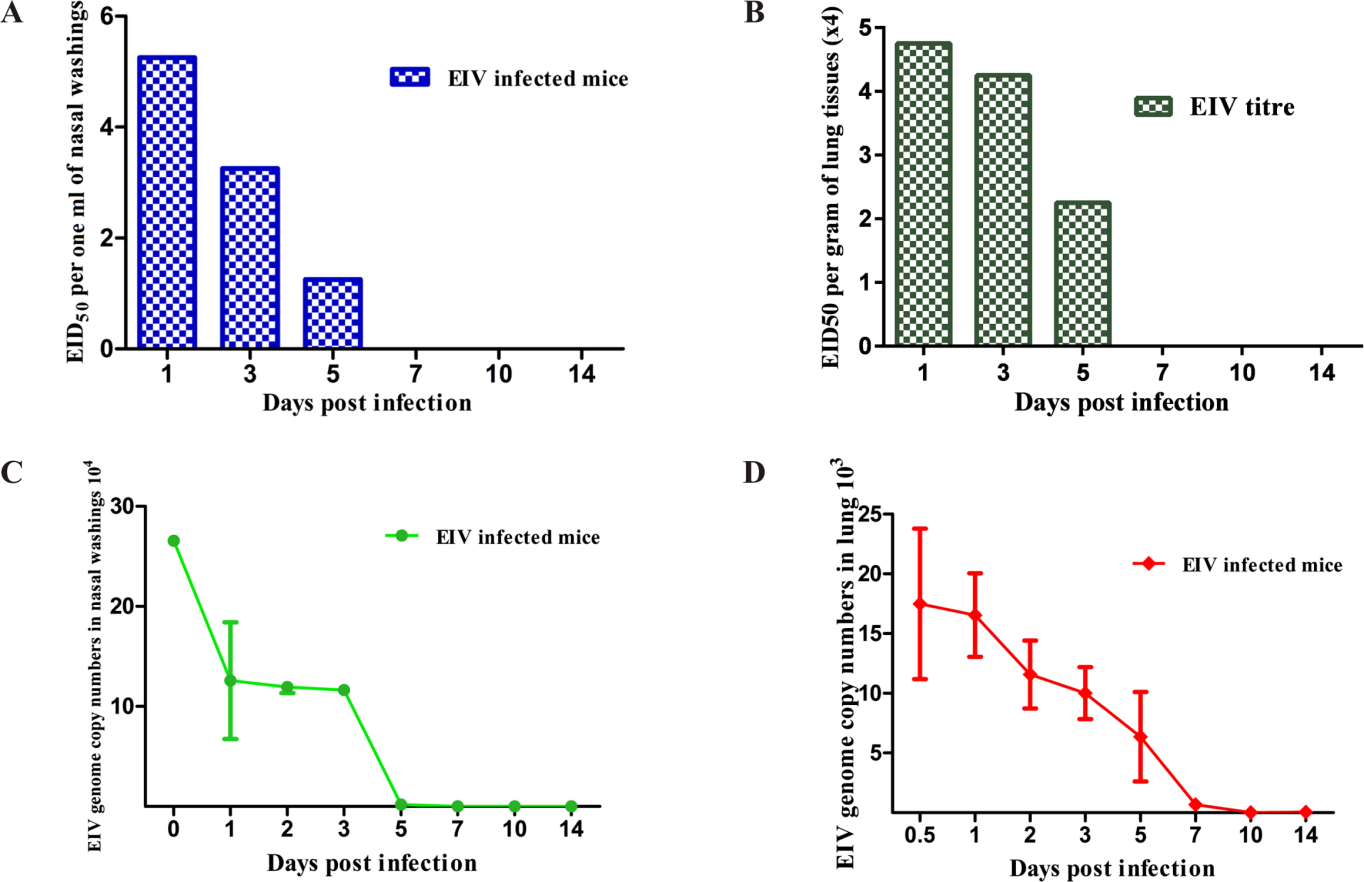
EIV replication kinetics. **A:** Quantification and duration of EIV shedding through mice nostril (per ml of nasal washings) following experimental infection with EIV. **B:** Replication kinetics of EIV in mice lung (per gram of lung tissues) following experimental infection in BALB/c mice. **C and D:** Quantification of EIV genome copy numbers in nasal washings (140 μl) (n = 3) and lung tissues (25mg) (n = 6) following experimental EIV infection in BALB/c mice (n = 6).

Shedding of EIV from the nasopharynx of infected mice was noted till 5 dpi and a peak virus titre (at 1 dpi) of 10^5.25^ EID_50_/ml was observed in nasal washings (Fig 8A). The virus titre reduced to 10^1.25^ EID_50_/ml by 5 dpi. In subsequent days no virus shedding was observed in nasal washings (S4 Table). EIV persisted in lung tissue up to 5 dpi, where in titres (quantified per gram of lung tissue) were 4X10^4.75^ EID_50_ at1 dpi, 4X10^4.25^ EID_50_ at 3 dpi and 4X10^2.25^ EID_50_ at 5 dpi (Fig 8B). Thereafter no EIV could be isolated (S5 Table).

### Quantification of EIV nucleic acid by qRT-PCR

The amplification plots of the qRT-PCR were observed to be smooth and linearity of the standard curves was perfectly good in triplicate test. The amplification efficiency of the assay was >96.0% and correlation coefficient (R^2^) was 0.98. qRT-PCR results revealed EIV shedding in nasal wash (140 μl) at much higher rates in infected mice up to 5 dpi with mean Ct values of 21.70±0.11, 22.5±0.07 and 22.54±0.05 at 1, 2 and 3 dpi, respectively. Mean Ct value increased to 29.01±0.27 at 5 dpi. The copy numbers of influenza virus observed in mice nasal washings at 3 and 5 dpi ranged from 1.12 x 10^5^ to 1.2 x 10^5^ and 1.5 x 10^3^ to 2.2 x 10^3^, respectively (Fig 8C) (S6 Table). Mean Ct value of infected mice lung tissues (25 mg) were 25.92±0.34, 26.52±0.34 and 28.72±1.02 at 1, 3 and 5 dpi, respectively. Maximum viral genome copy numbers were detected in infected mice up to 7 dpi with copy numbers ranging from 5.4 x 10^3^ to 3.8 x 10^4^, 3.5 x 10^3^ to 1.8 x 10^4^ and 4.5 x10^2^ to 2.2 x 10^4^ at 1, 3 and 5 dpi, respectively (Fig 8D). On subsequent days, copy numbers were insignificant (S7 Table).

## Discussion

Murine model has oftenly been considered as a best choice amongst small animal model to determine pathology and immune response to influenza virus infections as also for preliminary assessment of human and avian influenza vaccines [43–46]. Pathology of EIV infection has been studied to a greater extent in the natural host [34, 47] where as Castleman *et al*. (2010), [25] while, comparing pathology of H3N8 of canine and equine origin in mice, inoculated an old lineage isolate from equines *viz*. eq/KY/91 (American lineage virus) and observed mild lesions in lung parenchyma. Further, eq/ROM/80, eq/GA/81 and eq/NM/03 H3N8 virus infection in BALB/c mice revealed replication of EIVs in mice respiratory tract [26]. A 6:2 reassortant ca eq/GA/81 live virus vaccine showed protective immune response in mice following homologous and heterologous strain wild virus challenge [27]. However, data regarding pathological features of EIV (H3N8) in small animal model is lacking and earlier studies have focused on virus kinetics and serological responses. Thus aim of the present study was to evaluate the pathology of EIV in BALB/c mice and to assess their suitability for studying disease dynamics.

Experimental EIV inoculation in mice induced severe form of acute respiratory disease with primary involvement of upper and lower respiratory tract. Disease progression and course of the EI infection in the current study with mice was brisk, simulating to the rapid onset of the disease in equines including sero-conversion and shedding of virus through nostrils. Findings of gross and histological lesions induced by EIV in mice were also comparable to EI infection in equines. Display of clinical signs *viz*. respiratory distress, forced expiration, ruffled coat, decreased activity, crouching at the corners appear to have occurred as a result of initiation of lesions in nasal turbinates, trachea and lungs (bronchi and bronchioles).

Reduction in the body weight is considered as important pathologic feature of influenza infection in mice [48–50]. In agreement with the previous findings, mice in the present study displayed a maximum of 5–6% weight reduction between 2 to 8 dpi which appears to be associated with development of lung lesions characterized by bronchitis, bronchiolitis and interstitial pneumonia and simultaneous virus recovery and its demonstration. Development of similar clinical signs with significant body weight reduction after influenza virus infection has also been observed in PR8 (mice adapted H1N1strain), seasonal H1N1 influenza, H3N2 (A/Beijing/89), H5N1 (A/Hong Kong/97) and H9N2 (A/chicken/Hong Kong/97) virus infection in mice [51–55]. Castleman *et al*. (2010) [25] reported clinical signs of disease at 2–5 dpi with 5% reduction in body weight at 5 dpi after experimental infection with canine/FL/04 (H3N8) in mice; however, mice inoculated with American lineage (eq/KY/91), predivergent (eq/ROM/80 and eq/GA/81) and Clade 2 (eq/NM/03) EIVs did not show any clinical signs and had no reduction in body weight [25, 26]. Reduction in body weight of mice in current experiment might be due to difference in dose of virus used for inoculation. No mortality and systemic spread of virus could be observed in any of the mice in present investigation with EIV.

Increased LDH level in serum from 1 to 10 dpi commensurate with the inflammatory changes observed in lungs. Similar changes in the LDH level in humans infected with LPAI H7N9 [56] and brochio-alveolar lavage in mice after infection with H1N1, WSN/33 viruses [57, 58] have been observed. Inflammation and cell damage of lung tissue releases LDH from cytoplasm to extracellular spaces and flows back across a alveolo-capillary membrane in to blood.

In all infected mice, gross and histopathological lesions were confined to respiratory tract including nasal mucosa, trachea and lungs between 2 and 10 dpi and comprised of congestion and gray discoloration of whole lung parenchyma with small focal areas of consolidation and red hepatization. Pulmonary hepatization has been reported by Muranaka *et al*. (2012) [34] in equines after experimental infection with A/equine/Ibaraki/07 (H3N8; eq/IB/07) (Table 1). Severe lung consolidation has been described as a pathological feature of influenza virus infection caused by the 1918 pandemic H1N1 virus and H5N1 viruses in humans [59, 60] as well as in animal models [61, 62].

Histological lesions in the nasal turbinate first appeared at 2 dpi and remained till 5 dpi while tracheal changes began as early as 12 hpi. Equines experimentally infected with eq/IB/07 exhibited similar degenerative lesions in the nasal turbinate and trachea [34]. Pandemic influenza virus infection in humans [63], H1N1 and H3N2 in guinea pigs [64], classical swine H1N1 in ferrets and mice [65], HPAI H5N1 in mice [48] resulted in comparable lesions and antigen distribution as observed in our studies. Abundant EIV antigen could be detected in nasal turbinate, tracheal, bronchial and bronchiolar epithelial cells, alveoli and interstitial macrophages which was in agreement with infection with H1N1 & HPAI H5N1 in human [66] and pandemic swine H1N1 in pigs [67].

Lungs showed bronchitis, bronchiolitis and interstitial pneumonia in histopathology and EIV antigen localization could be demonstrated with immunohistochemistry. Further, budding of virions from surface epithelial cells of bronchi by TEM, supported by virus isolation and qRT-PCR confirmed replication of EIV. In a previous study in mice infected with eq/KY/91 tracheal and lung lesions were mild which was perhaps due to the fact that a laboratory adapted virus at high passage level from older lineage was used [25]. Our findings with regard to antigen distribution and ultra structural changes are in conformity with the observation of Castleman et al. (2010) [25] in mice and canines infected with A/canine/Florida/04. Experimental infection in equines with eq/IB/07 produced lesions of bronchopneumonia in second phase of febrile illness, while horses naturally infected with A/equine/Sydney/07 showed lesions of proliferative tracheitis, bronchointerstitial pneumonia and bacterial bronchopneumonia (Table 1) [34, 42]. Lesions in lungs of mice were comparable to host species with minor differences such as presence of more interstitial, alveolar, bronchiolar changes and early inflammatory cellular responses in mice, which can be explained in relation to differences in localization of receptors for binding of HA molecule for various species. EIV predominantly bind to host receptors with oligosaccharrides ending with sialic acid α-2,3-galactose which are predominantly located in the upper respiratory tract and to a lesser extent in the bronchial epithelium of equines [68] as compared to tracheal ciliated epithelial cells, type II alveolar epithelial cells [25] along with bronchi, bronchiolar epithelial cells and alveoli [69] in mice, which explains the difference in pathology., Various researchers working with H3N2 in canine, HPAI H5N1 in human and mice, pandemic H1N1 in mice, pandemic swine H1N1 in pigs, H1N1 and HPAI H5N1 in human and novel swine H1N1 in pigs have reported lung lesions of varying intensity [17, 54, 66, 67, 70–74].

Duration of virus shedding through nostrils in present studies (up to 5 dpi) is similar to experimental EIV infection in equines where virus could be recovered from nasal washings for a period of 5–7 dpi [34–36, 75, 76]. Similarly, from mice inoculated with eq/ROM/80, eq/GA/81 and eq/NM/03 isolates, infectious EIV was isolated from lungs and nasal turbinate up to 7 dpi [26]. Although, titre of recovered virus from mice nasal turbinate and lung in present investigation was slightly lower than previous studies yet virus multiplications in respiratory tract was adequately demonstrated by budding and release of virion particles in TEM. The RNA copy numbers in lung tissue were significantly higher in infected mice and persistence of virus in lung was detected up to 7 dpi.

It can thus be concluded from the present investigations that EIV {eq/J-K/08 (H3N8)} belonging to clade 2 of Florida sub lineage infect BALB/c mice and lead to rapid onset of respiratory illness with clinical signs, seroconversion, substantial virus shedding associated with respiratory illness characterized by gross and histopathological lesions in upper respiratory tract and lungs, no mortality and slow recovery. The pathology of EI in mouse model has satisfactory resemblance to the disease in natural host and studies garner sufficient evidence to qualify BALB/c mice as a model for studying EIV.

## Conclusions

In conclusion, this BALB/c mouse model for EI infection offers an attractive valuable tool for studying the interaction of EIV and host immune system, dissecting pathophysiology and molecular pathogenesis of EIV.

## Supporting Information

S1 Table Percent changes in body weight of control and EIV infected mice (n = 6). (DOC)

S2 Table Humoral immune response: Haemagglutination inhibition (HAI) assay results of BALB/c mice following infection with EIV. (DOC)

S3 Table Serum biochemistry: LDH levels of BALB/c mice after infection with EIV (pooled serum samples from each group, n = 6). (DOC)

S4 Table EIV titre in nasal washings from EIV infected mice at various intervals (pooled nasal washings from each group, n = 6). (DOC)

S5 Table Residual EIV titre in lungs of mice at various intervalsafter infection with EIV (pooled lung tissues from each group, n = 6). (DOC)

S6 Table Shedding of EIV in nasal washings (in terms of Ct values and viral RNA copy number) at various intervals after inoculation with EIV (n = 3). (DOC)

S7 Table Comparison of residual EIV in lung tissues of group A and group B mice (in terms of Ct values and viral RNA copy number) at various intervals after challenge with EIV (n = 6). (DOC)
