# Supplementary material for: Pathology of Equine Influenza virus (H3N8) in Murine Model
Source: PLoS One. 2015 Nov 20;10(11):e0143094. doi: 10.1371/journal.pone.0143094 (PMC4654517; doi:10.1371/journal.pone.0143094)
Supplement: S1 Table — (DOC) [file pone.0143094.s001.doc]

**S1 Table**. Percent changes in body weight of control and EIV infected mice (n=6)

| **Days post infection** | **EIV infected mice (± SEM)** | **Negative control mice(± SEM)** |
| --- | --- | --- |
| 1 | -3.64±0.08 | 0.11±0.02 |
| 2 | -4.62±0.11 | 0.09±0.11 |
| 3 | -4.78±0.19 | 0.17±0.09 |
| 4 | -5.6±0.09 | 0.58±0.17 |
| 5 | -6.34±0.21 | 0.43±0.14 |
| 6 | -5.87±0.14 | 0.52±0.24 |
| 7 | -6.15±0.18 | 0.76±0.18 |
| 8 | -5.37±0.12 | 0.91±0.09 |
| 9 | -4.93±0.19 | 1.54±0.15 |
| 10 | -5.15±0.06 | 1.66±0.21 |
| 11 | -5.01±0.18 | 1.74±0.16 |
| 12 | -4.68±0.08 | 2.04±0.14 |
| 13 | -4.57±0.16 | 2.44±0.22 |
| 14 | -3.91±0.11 | 2.57±0.13 |
