# Supplementary material for: Pathology of Equine Influenza virus (H3N8) in Murine Model
Source: PLoS One. 2015 Nov 20;10(11):e0143094. doi: 10.1371/journal.pone.0143094 (PMC4654517; doi:10.1371/journal.pone.0143094)
Supplement: S2 Table — (DOC) [file pone.0143094.s002.doc]

**S2 Table. Humoral immune response: Haemagglutination inhibition (HAI) assay results of BALB/c mice following infection with EIV**

| **Days post infection** | **Mean HAI titre (± SEM) (n=6)** |
| --- | --- |
| 0 | 0 |
| 1 | 0 |
| 2 | 0 |
| 3 | 0 |
| 5 | 17.33±3.21 |
| 7 | 37.33±5.33 |
| 10 | 85.33±13.49 |
| 14 | 106.67±13.49 |
