# Supplementary material for: Pathology of Equine Influenza virus (H3N8) in Murine Model
Source: PLoS One. 2015 Nov 20;10(11):e0143094. doi: 10.1371/journal.pone.0143094 (PMC4654517; doi:10.1371/journal.pone.0143094)
Supplement: S3 Table — (DOC) [file pone.0143094.s003.doc]

**S3 Table. Serum biochemistry: LDH levels of BALB/c mice after infection with EIV (pooled serum samples from each group, n=6)**

| **Days post infection** | **LDH level in different group of mice (IU/L)** | |
| --- | --- | --- |
|  | **EIV infected mice** | **Negative control mice** |
| 0 | 4413 | 422 |
| 1 | 4018 | 547 |
| 2 | 6344 | 594 |
| 3 | 5048 | 539 |
| 5 | 3944 | 498 |
| 7 | 3662 | 565 |
| 10 | 3256 | 486 |
| 14 | 1049 | 511 |
