# Supplementary material for: Pathology of Equine Influenza virus (H3N8) in Murine Model
Source: PLoS One. 2015 Nov 20;10(11):e0143094. doi: 10.1371/journal.pone.0143094 (PMC4654517; doi:10.1371/journal.pone.0143094)
Supplement: S4 Table — (DOC) [file pone.0143094.s004.doc]

**S4 Table. EIV titre in nasal washings from EIV infected mice at various intervals (pooled nasal washings from each group, n=6)**

| **Days post infection** | **EID_50_ per ml of nasal washings** |
| --- | --- |
|  | 10 ^5.25^ |
| 1 |  |
| 3 | 10 ^3.25^ |
| 5 | 10 ^1.25^ |
| 7 | Not detected |
