# Supplementary material for: Pathology of Equine Influenza virus (H3N8) in Murine Model
Source: PLoS One. 2015 Nov 20;10(11):e0143094. doi: 10.1371/journal.pone.0143094 (PMC4654517; doi:10.1371/journal.pone.0143094)
Supplement: S5 Table — (DOC) [file pone.0143094.s005.doc]

**S5 Table. Residual EIV titre in lungs of mice at various intervals after infection with EIV (pooled lung tissues from each group, n=6)**

| **Days post infection** | **EID_50_ per gram of lung tissues** |
| --- | --- |
| 1 | 4x10 ^4.75^ |
| 3 | 4x10 ^4.25^ |
| 5 | 4x10 ^2.25^ |
| 7 | Not detected |
