# Supplementary material for: Pathology of Equine Influenza virus (H3N8) in Murine Model
Source: PLoS One. 2015 Nov 20;10(11):e0143094. doi: 10.1371/journal.pone.0143094 (PMC4654517; doi:10.1371/journal.pone.0143094)
Supplement: S6 Table — (DOC) [file pone.0143094.s006.doc]

**S6 Table. Shedding of EIV in nasal washings (in terms of Ct values and viral RNA copy number) at various intervals after inoculation with EIV (n=3)**

| **Days post infection (days)** | **Ct value (± SEM)** | **Copy numbers (± SEM)** |
| --- | --- | --- |
| 12 hours | 21.25 | 265532.2±975.3 |
| 1 | 21.70±0.11 | 125803.1±58381.8 |
| 2 | 22.5±0.07 | 119270±5845.9 |
| 3 | 22.54±0.05 | 116421.2±3802.7 |
| 5 | 29.01±0.27 | 1899.6±329.9 |
| 7 | 37.65 | 3.7±0.2 |
| 10 | 37.71 | 6.6±0.5 |
| 14 | 38.05±0.19 | 5.8±0.7 |
