# Supplementary material for: Pathology of Equine Influenza virus (H3N8) in Murine Model
Source: PLoS One. 2015 Nov 20;10(11):e0143094. doi: 10.1371/journal.pone.0143094 (PMC4654517; doi:10.1371/journal.pone.0143094)
Supplement: S7 Table — (DOC) [file pone.0143094.s007.doc]

**S7 Table. Comparison of residual EIV in lung tissues of group A and group B mice (in terms of Ct values and viral RNA copy number) at various intervals after challenge with EIV (n=6)**

| **Days post infection (days)** | **Ct values (± SEM)** | **Copy numbers (± SEM)** |
| --- | --- | --- |
| 12 hours | 26.20±0.58 | 17473.6±6296 |
| 1 | 25.92±0.34 | 16541.8±3492.8 |
| 2 | 26.38±0.47 | 11560.3±2846.1 |
| 3 | 26.52±0.34 | 10004±2174.9 |
| 5 | 28.72±1.02 | 6345±3743.3 |
| 7 | 31.63±1.04 | 677.9±378.7 |
| 10 | 37.4±0.32 | 5.2±1.3 |
| 14 | 36.5±0.58 | 13.8±5.6 |
